# Genome-wide identification and expression pattern analysis of R2R3-MYB transcription factor gene family involved in puerarin biosynthesis and response to hormone in *Pueraria lobata var. thomsonii*

**DOI:** 10.1186/s12870-023-04115-z

**Published:** 2023-02-22

**Authors:** Zhengdan Wu, Wendan Zeng, Changfu Li, Jihua Wang, Xiaohong Shang, Liang Xiao, Sheng Cao, Yansheng Zhang, Shiqiang Xu, Huabing Yan

**Affiliations:** 1 Cash Crops Research Institute, Guangxi Academy of Agricultural Sciences, Nanning, 530007 China; 2 Shanghai Key Laboratory of Bio-Energy Crops, Research Center for Natural Products, Plant Science Center, School of Life Sciences, Shanghai University, Shanghai, 200444 China; 3 Guangdong Provincial Key Laboratory of Crops Genetics and Improvement, Crop Research Institute, Guangdong Academy of Agriculture Sciences, Guangzhou, 510640 China

**Keywords:** *Pueraria lobata var. thomsonii*, R2R3-MYB TFs, Expression patterns, Puerarin biosynthesis, Hormone response

## Abstract

**Background:**

R2R3-MYB transcription factors regulate secondary metabolism, stress responses and development in various plants. Puerarin is a bioactive ingredient and most abundant secondary metabolite isolated from *Pueraria lobata*. The biosynthesis of puerarin proceeds via the phenylpropanoid pathway and isoflavonoids pathway, in which 9 key enzymes are involved. The expression of these structural genes is under control of specific *PtR2R3-MYB* genes in different plant tissues. However, how *PtR2R3-MYB* genes regulates structural genes in puerarin biosynthesis remains elusive. This study mined the *PtR2R3-MYB* genes involved in puerarin biosynthesis and response to hormone in *Pueraria lobata var. thomsonii*.

**Results:**

A total of 209 PtR2R3-MYB proteins were identified, in which classified into 34 subgroups based on the phylogenetic topology and the classification of the R2R3-MYB superfamily in *Arabidopsis thaliana*. Furtherly physical and chemical characteristics, gene structure, and conserved motif analysis were also used to further analyze *PtR2R3-MYBs*. Combining puerarin content and RNA-seq data, speculated on the regulated puerarin biosynthesis of *PtR2R3-MYB* genes and structural genes, thus 21 *PtR2R3-MYB* genes and 25 structural genes were selected for validation gene expression and further explore its response to MeJA and GSH treatment by using qRT-PCR analysis technique. Correlation analysis and cis-acting element analysis revealed that 6 *PtR2R3-MYB* genes (*PtMYB039*, *PtMYB057*, *PtMYB080*, *PtMYB109*, *PtMYB115* and *PtMYB138*) and 7 structural genes (*PtHID2*, *PtHID9*, *PtIFS3*, *PtUGT069*, *PtUGT188*, *PtUGT286* and *PtUGT297*) were directly or indirectly regulation of puerarin biosynthesis in ZG11. It is worth noting that after MeJA and GSH treatment for 12–24 h, the expression changes of most candidate genes were consistent with the correlation of puerarin biosynthesis, which also shows that MeJA and GSH have the potential to mediate puerarin biosynthesis by regulating gene expression in ZG11.

**Conclusions:**

Overall, this study provides a comprehensive understanding of the *PtR2R3-MYB* and will paves the way to reveal the transcriptional regulation of puerarin biosynthesis and response to phytohormone of *PtR2R3-MYB* genes in *Pueraria lobata var. thomsonii*.

**Supplementary Information:**

The online version contains supplementary material available at 10.1186/s12870-023-04115-z.

## Background

The genus Pueraria belonging to the Leguminosae family was originated in Asia and comprises of more than 20 species [32]. *Pueraria lobata* is a well-known traditional Chinese medicinal herb and widely grown in China for a long time. The roots of both *Pueraria lobata* (hereinafter abbreviated as *P. lobata*) and *Pueraria lobata var. thomsonii* (hereinafter abbreviated as *P. thomsonii*) have long been used for treating fever, toxicosis, indigestion, and liver damage from alcohol abuse in traditonal Chinese medicine [27, 38, 48, 51]. Puerarin is an important bioactive constituent, in which extracted from the dry roots of this plant [15, 38]. As the standard to evaluate the quality of *P. thomsonii*, the Chinese Pharmacopoeia (2020 edition) specifies that the content of puerarin shall not be less than 0.30%. However, the puerarin content of the cultivated *P. thomsonii* hardly reaches the Standard of Chinese Pharmacopoeia, which significantly restrains its pharmaceutical prospects.

Puerarin are classified as isoflavones and biosynthesized from L-phenylalanine through a set of reaction that is catalyzed by phenylalanine ammonialyase (PAL), cinnamate 4-hydroxylase (C4H), 4-coumarate-CoA ligase (4CL), chalcone synthase (CHS), chalcone isomerase (CHI), chalcone reductase (CHR) in phenylpropanoid pathway and 2-hydroxyisoflavanone (IFS), 2-hydroxyisoflavanone dehydratase (HID), UDP-glucosyltransferases (UGT) in isoflavonoids pathway [43]. At the transcription level, isoflavone biosynthesis is mainly regulated by structural genes (SGs) and transcription factors (TFs) [12, 15].

MYB is a large transcription factor family in higher plants and can be divided into 1R-MYB, R2R3-MYB, 3R-MYB, and 4R-MYB subfamilies according to the number of highly conserved N-terminal MYB DNA-binding domains repeats [9, 44]. R2R3-MYB TFs regulated secondary metabolism and have been demonstrated as key factors for the regulation of flavonoid biosynthesis [28, 45]. In Arabidopsis, a total of 126 R2R3-MYBs have been divided into 25 subfamilies based on the conservation of DNA binding domain and amino acid motifs, of which subfamily S3, S4, S5, S6, S7 and S13 were involved the regulation of the phenylpropanoid pathway [9]. Among the soybeans with the closest relatives of *P. thomsonii*, *GmMYB176*, *GmMYBJ3*, *GmMYB29* mediated isoflavone biosynthesis by regulating the expression of SGs [3, 46, 50]. Over-expression of *PlMYB1* in *Arabidopsis thaliana* significantly increased the accumulation of anthocyanins in leaves and proanthocyanidins in seeds, by activating *AtDFR*, *AtANR*, and *AtANS* genes [39].

MYB participate in the abiotic stress responses through mediating biosynthesis and accumulation of secondary metabolites [31]. The *PcMYB10*-*PcMYC2* molecular complex is likely involved in the regulation of methyl jasmonate (MeJA) -induced flavonoid biosynthesis at the transcript level in pear calli [34]. The puerarin content of *P. lobata* callus cells suspension treatment with different concentrations of MeJA was also increased significantly, up to 25 times [11]. The study by Li et al. [26] also confirmed the puerarin content was increased at 24 h and 48 h in *P. lobata* cell suspension cultures after MeJA treatment. Flavonoids have strong antioxidant activity, which can help plants to eliminate reactive oxygen species [33]. Exogenous GSH, an antioxidant molecule that reduces oxidative stress, increased the contents of flavonoids related metabolites in maize [42]. GSH-induced enhancement in Cd tolerance was closely associated with the upregulation of transcripts of several transcription factors such as MYB1 TF- AIM1 and R2R3-MYB TF- AN2 in tomato [14].

Although there has been extensively studied about the MYB genes in higher plants, almost no MYB genes studies have been reported in *P. thomsonii*. In present study, a total of 209 *PtR2R3-MYB* genes were identified. Furthermore, the physical and chemical characteristics, subcellular localization, gene structure, conserved motif, chromosomal location, phylogenetic relationships were performed. To mine out *PtR2R3-MYB* genes and SGs link with puerarin biosynthesis, we analyzed the correlation between puerarin content and expression pattern of *PtR2R3-MYB* genes and SGs of puerarin biosynthesis by RNA-seq and qRT-PCR. In order to further explore the underlying correlation between the *PtR2R3-MYB* genes and SGs, the analysis of expression patterns under MeJA and GSH treatment were performed by qRT-PCR. Moreover, we analyzed the cis-element of genes promoters to identify *PtR2R3-MYB* genes and SGs to determine direct or indirect regulatory relationships between them. In addition, we also determined the potential of hormones to regulate puerarin synthesis in puerarin. These finding indicated a feasible role for *PtR2R3-MYB* genes in puerarin biosynthesis and expanded our understanding of corresponding molecular mechanisms by PtR2R3-MYB-SGs model in modulating hormone response in *P. thomsonii*.

## Results

### Identification of R2R3-MYB genes in *P. thomsonii*

**Fig. 1:**
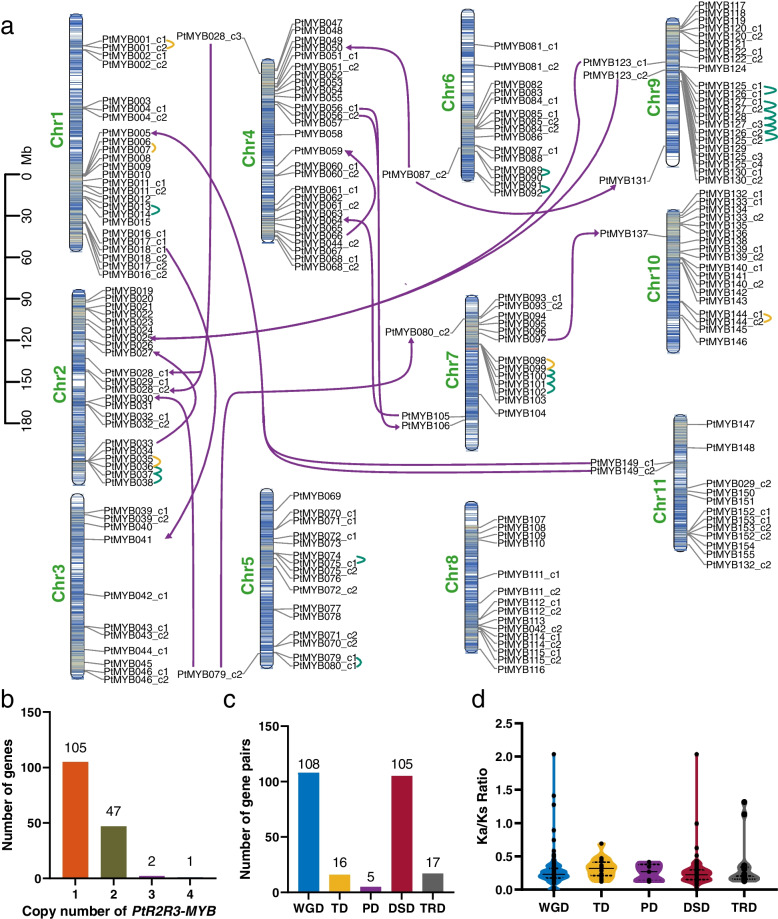
Chromosomal distribution of *R2R3-MYB* genes on 11 *P. thomsonii* chromosomes. **a** Distribution of *PtR2R3-MYB* genes in 11 chromosomes. Tandemly and proximal duplicated genes are marked with green and yellow lines, respectively. Transposed duplicated genes are marked with purple lines, arrow points to transposed genes. **b** Distribution of genes with different copy of *PtR2R3-MYBs*. **c** Distribution of gene pairs with different duplication model of *PtR2R3-MYBs*. **d** The Ka/Ks ratio distributions of gene pairs derived from different modes of duplication. The center line is the median; the lower and upper dotted line correspond to the first and third quartiles (25th and 75th percentiles). WGD, Whole-genome duplication; TD, Tandem duplication; PD, Proximal duplication; DSD, Dispersed duplication; TRD, Transposed duplication

According to the protein family database, the *P. thomsonii* genome contains 436 CDS sequences encoding MYB proteins and were divided into 4 classes which including 206 1R-MYB, 220 R2R3-MYB, 8 3R-MYB, 2 4R-MYB (Table S1). After redundancy removal, 209 *PtR2R3-MYB* CDS sequences were obtained. By Multiple Sequence Alignment, a total 209 *PtR2R3-MYB* genes were identified and we named them *PtMYB001_c1* to *PtMYB155* according to their chromosomal location (“c” stands for copy, Fig. 1a and Table S2). For *R2R3-MYB* genes in the *P. thomsonii*, we found 67.74% with one copy and 30.32% with two copies, only *PtMYB028* and *PtMYB127* have 3 copies and *PtMYB125* has 4 copies (Fig. 1b and Table S2).

The basic information of *PtR2R3-MYB* genes was analyzed and summarized in detail, including their protein sequences length, MW, pI, and subcellular localization. The lengths of the protein sequences of PtR2R3-MYB range from 182 to 1686 amino acids, and molecular weight vary from 20.87 kDa (*PtMYB119*) to 183.21 kDa (*PtMYB015*). Moreover, the theoretical isoelectric point (pI) ranged from 4.67 (*PtMYB005*) to 9.72 (*PtMYB121*). Some other parameters, such as instability index, aliphatic index and grand average of hydropathicity, were detailed in the Table S2. Subcellular localization prediction showed that all PtR2R3-MYB proteins were localized in the nucleus (Table S3). The results also suggest that a few PtR2R3-MYB may be dual-localized.

### Chromosomal location and duplication events of *PtR2R3-MYB* genes

The identified 209 *R2R3-MYB* genes were unevenly distributed on 11 chromosomes of *P. thomsonii* (Fig. 1a), indicating the diversification and complexity of the *PtR2R3-MYB* family. Chromosome 4 contained the largest number of *PtR2R3-MYB* genes (29 genes, ~ 13.88%), followed by chromosome 1 and 9 (25 genes, ~ 11.96%), and chromosome 3 contained the smallest number of PtR2R3-MYB genes (11 genes, ~ 5.26%).

Gene duplication events playing a crucial role in evolution and speciation of plants. To further investigate the evolution and duplication events of *PtR2R3-MYB* genes, we analyzed the types of duplication events. We identified 251 duplicated gene pairs that were classified into five different categories: 108 whole-genome duplicates (WGD duplicates, 43.0%), 16 tandem duplicates (TD, 6.4%), 5 proximal duplicates (PD, 2.0%), 17 transposed duplicates (TRD, 6.8%), and 105 dispersed duplicates (DSD, 41.8%) (Fig. 1c, Table S4).

To trace duplication time of *PtR2R3-MYB* duplicated gene pairs, we compared the Ka, Ks, Ka/Ks and duplication time distribution among different modes of gene duplication (Fig. 1d, Table S4). Higher Ka/Ks ratios and smaller Ks values were found for TD gene pairs, suggesting an ongoing and continuous process for TD and more rapid sequence divergence and stronger positive selection than genes originated through other duplication modes. Lower Ka/Ks ratios and largest Ks values were found for PD gene pairs, suggesting slower sequence divergence and stronger negative selection than other duplication modes.

### Phylogenetic trees and group classification of *R2R3-MYB* genes in *P. thomsonii*

**Fig. 2:**
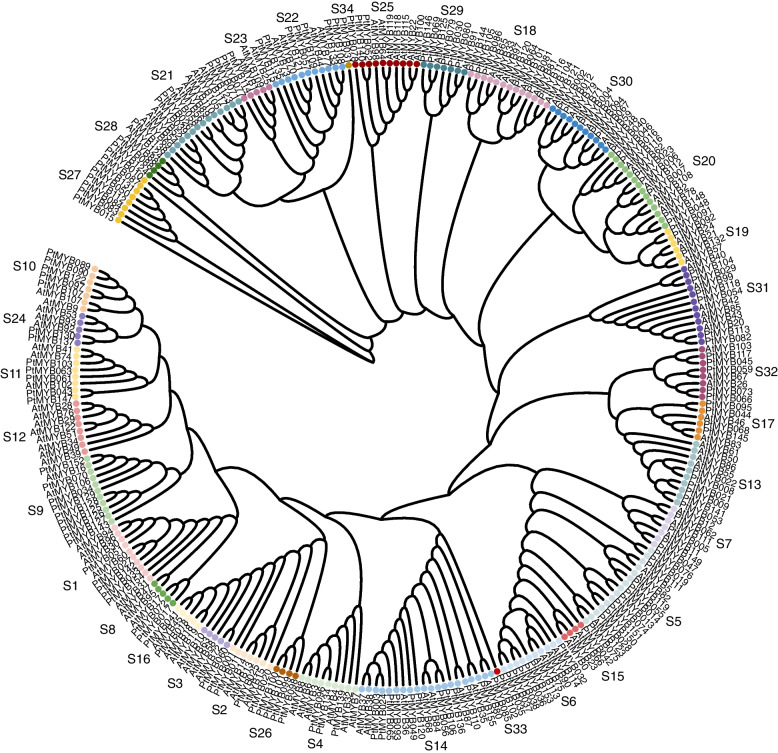
Phylogenetic tree of R2R3-MYB proteins. A Maximum Likelihood phylogenetic tree was constructed from 281 protein sequences including all R2R3-MYB proteins from *P. thomsonii* (155) and *A. thaliana* (126). Subgroups within each clade were given a different color; meanwhile, the same color indicates the genes are in the same subgroup

To understand the phylogenetic relationship and explore potential molecular function of the *R2R3-MYB* genes, phylogenetic analysis was performed using all R2R3-MYB full-length protein sequences from *P. thomsonii* (155 genes, multicopy genes are analyzed using only one sequence) and *A. thaliana* (126 genes). These 155 *PtR2R3-MYB* genes were divided into 34 subfamilies (S1 ~ S34) according to the topology of the tree and classification of the *AtMYB* superfamily (Fig. 2, Table S5). S5 and S14 had 12 members, which were the largest group, while S12 had 0 members, which was the smallest group.

### Conserved motif and structural analysis of *PtR2R3-MYBs*

**Fig. 3:**
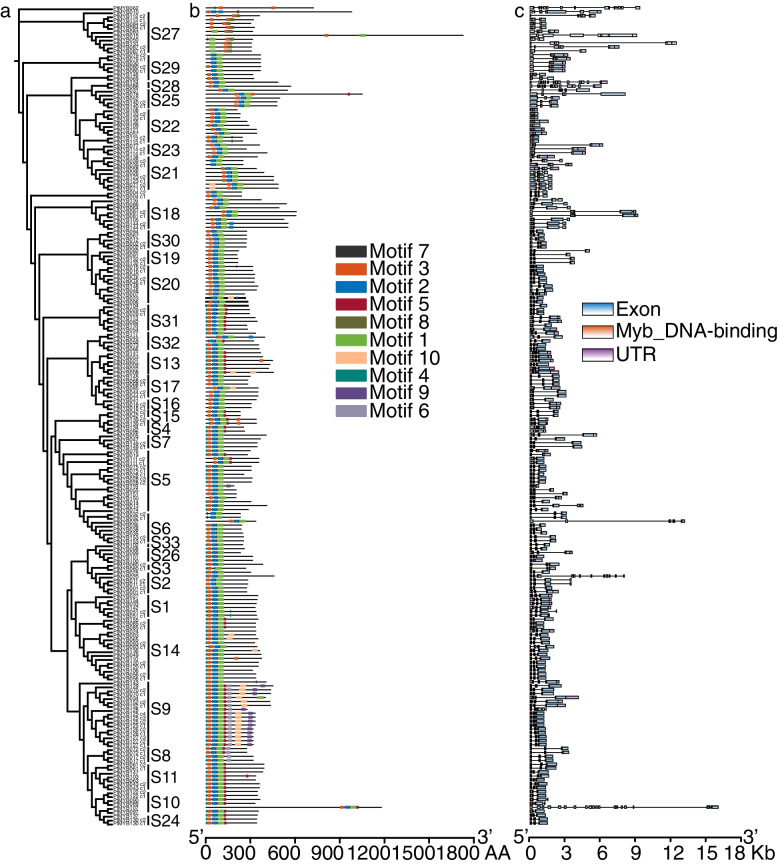
Phylogenetic analysis, conserved motifs and gene structure of *PtR2R3-MYB* genes. **a** Phylogenetic tree analysis derived from ML methods of PtR2R3-MYB proteins. Bootstrap values by using 1000 replicated are indicated at each node. Gene names are shown on the right. **b** Distribution of 10 conserved motifs within PtR2R3-MYBs. Differently colored rectangles represented different motifs. **c** Gene structure of PtR2R3-MYB genes. The untranslated region (UTRs), exons and introns region are represented by purple rectangles, blue rectangles, black lines, respectively. The MYB-DNA binding domain are indicated by yellow rectangles

To gain more insight into the evolution and structural diversity of the *PtR2R3-MYBs*, we analyzed conserved motif in the amino acid sequences using the MEME suit, a total of 10 distinct and highly conserved motifs were captured (Fig. 3b, Table S6). Motif 1, 2, 3 and 8 were identified as MYB domains, while other motifs (4, 5, 6, 7, 9 and 10) were function unknown (Table S6). Most *PtR2R3-MYB* subfamilies members were highly conserved in motif distribution pattern and contain motif 1, motif 2, motif 3, motif 7 (Fig. 3a and b). In contrast, a large proportion of motifs displayed specificity to different subfamilies, such as motif 6 was only present in S9 and S31 subfamily. Members of the subfamilies S9 contains 9 motif but lack motif 8, of which motif 9 and motif 10 are unique to this subfamily. Subfamily S27 contains fewer motifs and is generally composed of two motif identified as MYB domains and one motif 8 unique to this subfamily.

To further explore the structural diversity of *PtR2R3-MYB* genes, the intron-exon organization of each *PtR2R3-MYB* gene was analyzed (Fig. 3c). As shown in Fig. 3c, the exon numbers of *PtR2R3-MYB* genes varied from 1 to 19. We found that most of the *PtR2R3-MYB* genes had 3 exons (144/209), while those with 12 and 9 exons existed just one each. Additionally, we also incorporated *PtR2R3-MYB* genes exon-intron information into the phylogenetic tree mentioned above, it was noteworthy that there were 9 genes containing 1 exon and these 9 genes were clustered in the same subfamily S22. Members of the subfamily S17 and S23 contain 2 exons. Some members of the *PtR2R3-MYB* subfamily differ in the number of exons, but the closer the phylogenetic trees were, the greater the similarity of gene structure was.

### Puerarin contents and expression pattern of *PtR2R3-MYB* genes among different tissues and varieties in *P. thomsonii*

**Fig. 4:**
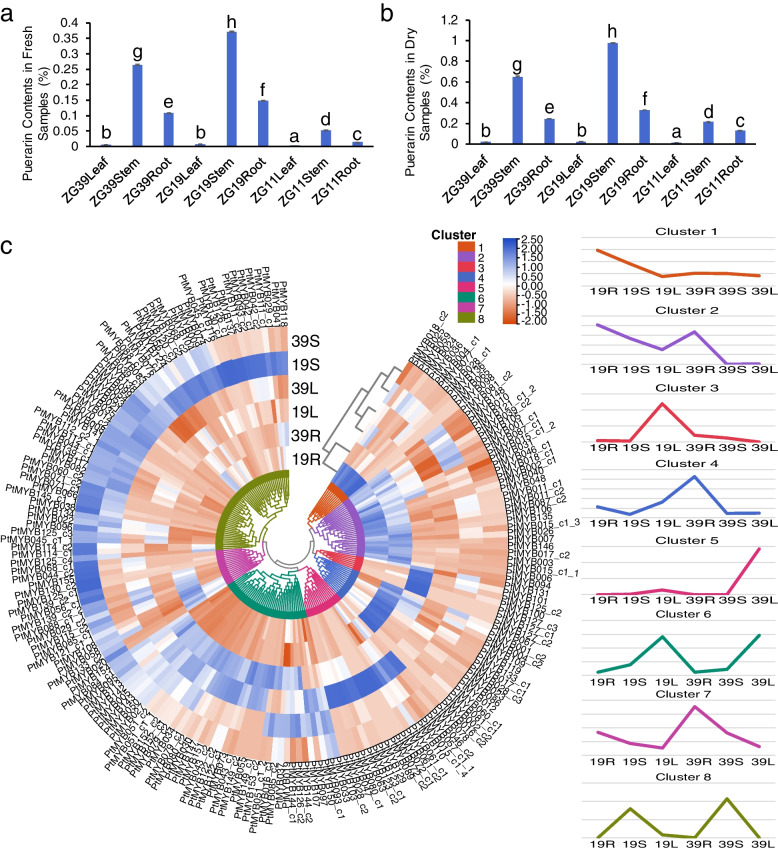
The puerarin content and expression patterns of *PtR2R3-MYB* genes in *P. thomsonii*. **a** Puerarin contents in fresh samples. The graph for each cell line shows the mean percentage of puerarin contents ± SD calculated from three independent experiments. Differences were considered significant at *p* value < 0.05. Different letters represent significant difference, the same letters represent no significant difference. The same below. **b** Puerarin contents in dry samples. **c** Heatmap of the *PtR2R3-MYB* genes transcriptome data in the various varieties and tissues. The gene names are shown on the out circles of heatmap. Transcript abundance is expressed in standardized log2 FPKM values. Genes highly or weakly expressed are colored blue and red, respectively. Clustering analysis was performed using Complete Linkage method with Pearson distances. The expression patterns of 8 clusters are shown on the right. 19R, ZG19Root; 19S, ZG19Stem; 19 L, ZG19Leaf; 39R, ZG39Root; 39S, ZG39Stem; 39 L, ZG39Leaf

Puerarin is the main active ingredient of *P. thomsonii*, which is highly valuable for its medicinal properties. We therefore determined the puerarin content in fresh and dried samples of roots, stems and leaves in ZG11, ZG19 and ZG39 by HPLC. As shown in Fig. 4a and b, the change trend of puerarin content in fresh samples and dried samples were relatively consistent. Among the different tissues, the change of puerarin content showed the same tendency in ZG11, ZG39 and ZG19, which is ZG19 > ZG39 > ZG11. In the same variety, the puerarin content in roots was greater than that of leaves and lower than that of stems.

The regulation of puerarin biosynthesis at transcription level may be operated by a series of transcript factors, especially for those R2R3-MYB genes. Transcriptome data of roots, stems, leaves in ZG19 and ZG39 was used to investigate expression profiles of *PtR2R3-MYB* genes (Fig. 4c). These expression patterns results clearly shown that these genes may have different roles at different tissues and varieties. The genes were classified into eight expression clusters, based on their distinct transcript patterns in various varieties and tissues. The 11 genes in cluster 1 were expressed in ZG19 higher than that in ZG39, in contrast to the 17 genes expression levels in cluster 7, which were lower expressed in ZG19 than in ZG39. Genes in cluster 2 (29) and cluster 8 (58) were expressed mainly in roots and stems, respectively. Genes in cluster 3 (7), cluster 4 (15) and cluster 5 (18) were predominately expressed in ZG19 leaf, ZG39 root and ZG39 leaf, respectively. Forty-two genes in cluster 6 were most highly expressed in leaves, with lower levels observed in roots.

Through Spearman correlation coefficient analysis, we found that the puerarin content was negatively and positively correlated with the expression levels of members of *PtR2R3-MYB* cluster 5 and 8, respectively (Fig. 4c, Table S7), indicating that those members may contribute to puerarin biosynthesis. Notably, expression levels of members of subfamily S5, S6, S7, S8, S23 and S31 were significantly correlated with puerarin content.

### Validation of *PtR2R3-MYBs* and SGs from RNA-seq data by qRT-PCR

**Fig. 5:**
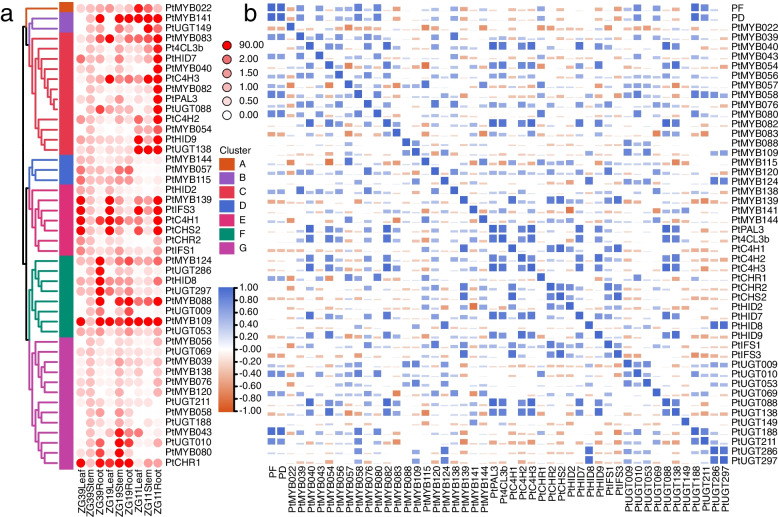
Expression pattern and correlation analysis of *PtR2R3-MYB*s and SGs in different tissues and varieties. **a** Heatmap showing hierarchical clustering analysis of *PtR2R3-MYB* genes and SGs across different tissues including leaves, stems and roots in ZG11, ZG39 and ZG19. The color scale is shown on the top right. The different colors represent the clustering of different group on middle right. **b** Correlative analysis between expression level of *PtR2R3-MYB* genes, SGs and puerarin contents in roots, stems, leaves of ZG11, ZG39 and ZG19. *r* > 0.5 indicated a positive correlation; *r* < − 0.5 indicates a negative correlation, the same below. The color scale is shown on the lower left

We also analyzed the expression patterns of 482 SGs involved in puerarin biosynthesis which were identified according to the genome annotation file (Table S8, [38]). We selected *PtR2R3-MYB* genes and SGs with significant correlation (*r*_s_^2^ > 0.64, Table S8) between gene expression level and puerarin content to perform qRT-PCR validated transcriptome data in roots, stems and leaves of ZG11, ZG19 and ZG39. As shown in Fig. 5a and Table S17, 46 genes were divided into 7 clusters via Average method with Pearson distances. *PtMYB022* in cluster A was highly expressed in ZG11, in contrast, 3 genes in cluster D were low expressed. Genes in cluster B (2) and cluster F (8) were not expressed in leaves and highly expression in roots, respectively. Twelve genes in cluster C had high expression mainly in roots of ZG11. The genes of cluster E were mainly expressed in leaves in ZG39 and ZG19, but were highly expressed in leaves and roots in ZG11. The 13 genes of cluster G were mainly highly expressed in stems.

We also further analyzed the correlations of puerarin content and expression pattern of *PtR2R3-MYB* genes and SGs (Fig. 5b, Tables S9-S12). Expression pattern of *PtMYB022* in cluster A was significantly correlated with puerarin content in ZG19 (*r* = − 0.955, Table S12). Genes in cluster B and D were positively and negatively correlated with puerarin content in ZG11, respectively (Fig. 8, Table S10). The genes of cluster C showed clear correlation of expression with puerarin content in ZG39 and ZG19 (Tables S11 and S12). The expression levels of the genes in cluster E were correlated with puerarin content in roots, stems and leaves of same varieties, but not with the puerarin content among varieties. As shown in Fig. 5b, the results of correlation analysis showed that there was a positively correlations between genes expression level of cluster G and puerarin content among 9 samples, indicating that these genes might be involved in the regulation of puerarin synthesis among different tissues and varieties.

Moreover, expression of 6 *PtR2R3-MYB* genes (*PtMYB039*, *PtMYB057*, *PtMYB080*, *PtMYB109*, *PtMYB115* and *PtMYB138*) and 7 SGs (*PtHID2*, *PtHID9*, *PtIFS3*, *PtUGT069*, *PtUGT188*, *PtUGT286* and *PtUGT297*) correlated with puerarin content (*r* > o.8 or *r* < 0.8) in ZG11, indicated that these genes may regulated the accumulated of puerarin in ZG11.

### Expression analysis of *PtR2R2-MYB*s and SGs under exogenous hormone treatments by qRT-PCR

**Fig. 6:**
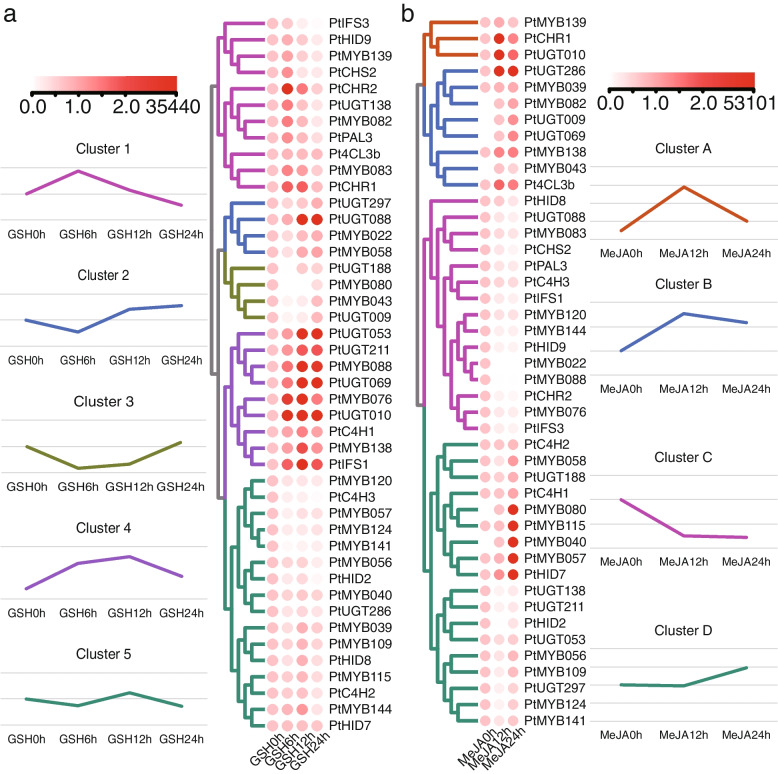
Heatmap of 44 genes following GSH and MeJA treatment in the root of ZG11. **a** GSH treatment. **b** MeJA treatment. Gene names are shown on the left. The color scale is shown on the left and right top of figure for GSH and MeJA treatment, respectively. The 44 genes clustered into 5 and 4 groups, based on their expression level after GSH and MeJA treatment, respectively

MYB was involved in abiotic stress responses by mediating the biosynthesis and accumulation of secondary metabolites [31]. MeJA and GSH are two effective elicitor of flavonoids biosynthesis. To investigate the role of the *PtR2R3-MYBs* and SGs in regulation of puerarin biosynthesis when pueraria adapts to various abiotic stresses, qRT-PCR was performed to examine the responses of 44 genes for exogenously added MeJA and GSH in the roots of ZG11. Forty-four genes were divided into 5 and 4 group after GSH (Fig. 6a) and MeJA (Fig. 6b) induction, respectively. It is worth noting that after MeJA and GSH treatment for 12–24 h, the expression changes of most candidate genes were consistent with the correlation of puerarin biosynthesis, that is, the expression of negatively regulated genes was repressed, and the expression of positively regulated genes was induced.

**Fig. 7:**
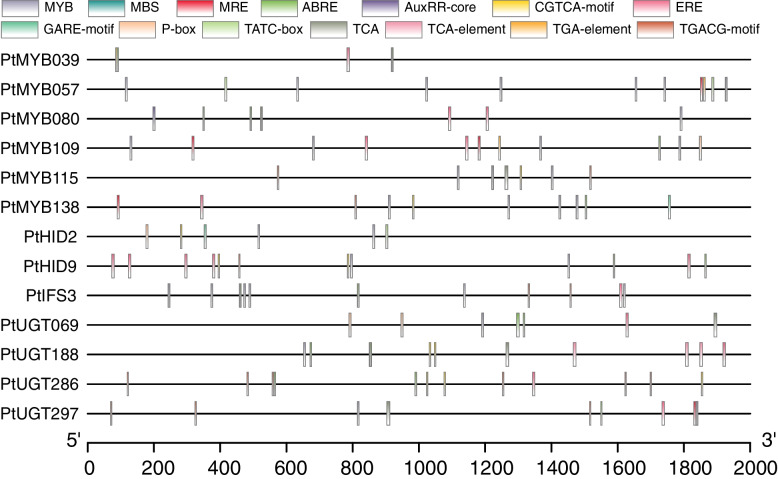
Distribution of cis-elements in the promoter regions of 13 candidate genes in ZG11. The locations of MYB DNA binding site and hormone-responsive cis-elements were confirmed using the PlantCARE database. Different cis-elements are represented by boxes of different colors

The promoter sequence of 13 puerarin biosynthesis significantly related genes also contains hormone responsive cis-elements in ZG11 (Fig. 7 and Table S13). Cis-elements were grouped into 6 classes according to their function, such as MeJA responsive elements (CGTCA-motif and CGTCA-motif), abscisic acid responsive elements (ABRE), ethylene responsive elements (ERE), salicylic acid responsive elements (TCA), gibberellin responsive elements (GARE-motif, P-box and TATC-box) and auxin responsive elements (AuxRR-core and TGA-element). Among the MeJA responsive elements, total of 11 CGTCA-motif and 15 TGACG-motif were identified in the promoter of 13 genes. The results suggested that puerarin biosynthesis related genes might have potential roles in various hormone signal responsiveness.

### Correlation analysis between *PtR2R3-MYBs* and SGs regulation of puerarin biosynthesis in ZG11

**Fig. 8:**
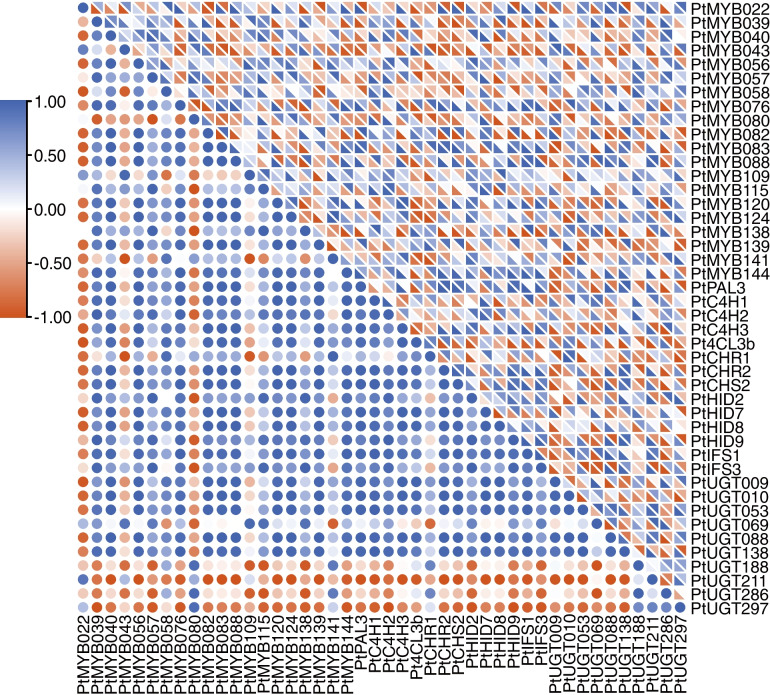
Correlation analysis of *PtR2R3-MYB* genes and SGs in ZG11. The circular at bottom left of graph represent correlation coefficient between *PtR2R3-MYB* genes and SGs in roots, stems, leaves of ZG11. The rectangles at top right of graph represent correlation coefficient between PtR2R3-MYB genes and SGs under MeJA (bottom left) and GSH (top right) treatment in ZG11. The color scale is shown on the left, blue represents high correlation, red represents low correlation

In order to identify downstream targets of *PtR2R3-MYBs*, we analyzed the expression level correlation between *PtR2R3-MYB*s and SGs in various tissues and under GSH and MeJA treatment of root in ZG11 (Fig. 8, Tables S10, S14 and S15). We found that the most genes correlated with the expression pattern of *PtMYB083* (*r* > 0.75), including *PtPAL3*, *PtCHR2*, *PtHID9*, *PtUGT138*, speculated that these genes were putative downstream targets of *PtMYB083*. *PtUGT297* and *PtUGT297* were putative downstream targets of *PtMYB139* and *PtMYB080*, *PtMYB141*, respectively. *PtUGT286* was positive-regulated by *PtMYB080*, but negative-regulated by *PtMYB076* and *PtMYB115* based on expression profile.

To further assess whether these genes might be direct targets, we analyzed the MYB binding sites of SGs promoter regions (Fig. 7, Table S13). As results shown in Fig. 8, the promoter of most structural genes contain MYB binding elements except *PtUGT286* and *PtHID7*. While the expression level of *PtUGT286* was significantly correlated with puerarin content (*r* = 0.994) and *PtMYB076* (*r* < − 0.5) and *PtMYB080* (*r* > 0.69), indicting that there is an indirect regulation between *PtUGT286* and *PtMYB076*, *PtMYB080*.

## Discussion

### Evolutionary analysis of the *PtR2R3-MYB* genes family

The MYB gene family is one of the largest TF families in plants, among which R2R3-MYB TFs are the most abundant type [16, 40]. With the genomes of more species completely sequenced, numerous R2R3-MYB genes have been identified, such as 126 R2R3-MYBs was identified in *A. thaliana* [9], 244 in soybean [6], 157 in maize [5] and 88 in *Oryza sativa* [19]. In this study, we systematically identified 209 R2R3-MYB members in *P. thomsonii* genome. *P. thomsonii* has markedly expanded R2R3-MYB gene families compared with the Arabidopsis, while the number MYB genes in plant did not depend entirely on genome size. The number of *PtR2R3-MYB* genes is approximately ~ 1.7 times more than that in Arabidopsis, which is close with the rate between the number of predicted genes in genome (*P. thomsonii*:45,270/Arabidopsis:23,498 ≈ 1.8) but not related to the genome size (*P. thomsonii*: 1.37Gb/ Arabidopsis: 125 Mb ≈ 11.2) [38, 47]. It indicates that the abundance of R2R3-MYB genes in *P. thomsonii* have expanded, that may be related with multiple gene duplication processes, including WGD, TD, PD, DSD and TRD events in *P. thomsonii*.

Variability in the number of R2R3-MYB genes might be attributed to the ploidy level of species and the number of gene duplication events in the different genomes evolution [37]. Lotus, maize, pear, cotton, click pea and potato also experienced duplication events that led to the expansion of the MYB gene family in their genomes, suggesting that gene duplication have promoted the expansion of the MYB gene family in different plants during the evolutionary process [4, 5, 10, 16, 37, 40]. The high number of genes in *P. thomsonii* is thought to be caused by two WGDs that occurred during *P. thomsonii* genome evolution [38]. The 108 (43% of all *PtR2R3-MYB* duplication events) *PtR2R3-MYB* duplication events can be explained by these two WGD events in this study (Table S4), one of which occurred after the divergence of *P. thomsonii* from *Glycine max* and was dated at 4.8 million years ago (Mya), another occurred early in the evolution of Leguminosae species (*Pisum sativum*, *Phaseolus vulgaris*, *Vigna unguiculate*, *Vigna angularis*, *Glycine max*), at 44.5 Mya. Genes may be duplicated by several mechanisms in addition to WGD, which have been collectively referred to as small scale duplications or single gene duplications [52]. In this study, 16 (6.4%) TD, 5 (2.0%) PD, 105 (41.8%) DSD and 17 (6.8%) TRD were identified in the *PtR2R3-MYB* gene duplication events (Fig. 1, Table S4), which contributed to the expansion of *PtR2R3-MYB* family. Taken together, our results suggested that the expansion of the *R2R3-MYB* gene family in *P. thomsonii* mainly arose from WGD and DSD events, accompanied by TRD, TD and PD events. Meanwhile, we noticed that TD and TRD gene pairs of *PtR2R3-MYB* exhibits a strong signature of positive selection during evolution of *P. thomsonii* compared with other duplication modes, which contributed to adaptive phenotypic evolution. The results also indicated that, among the duplication events in the *PtR2R3-MYB* gene family, the gene pairs that appeared to be derived from TD events occurred later than those that arose from other modes of duplication, suggesting slower sequence divergence at TD in *P. thomsonii*.

In this study, the expression levels of the identified tandem-duplicated genes were same and varied among tissues and variety, respectively (Fig. 4). For instance, *PtMYB013* and *PtMYB014* were a pair of tandem-duplicated genes, *PtMYB014* was highest expressed in stems of ZG39 and ZG19, but *PtMYB013* not expressed. We speculate that the reason may be *PtMYB013* achieving non-functionalization through silencing during evolution.

### *PtR2R3-MYB* genes play crucial roles in puerarin biosynthesis

Puerarin content is an important medicinal indicator that usually low in *P. thomsonii*. ZG39 had a puerarin content greater than ZG11 but less than ZG19 (Fig. 4). The large variation in puerarin concentrations among different varieties suggested that varieties selection is a key parameter for optimizing the puerarin concentration. Metabolomics studies also have shown that puerarin predominantly accumulated in *P. lotaba* roots and stems compared to leaves in young stage and higher in mature roots than in young roots [13, 26]. In the present study, mature stems and roots contained more puerarin than mature leaves, and stems were larger than roots. The puerarin was mainly found in the phloem and xylem in *P. lotaba* [7]. Roots and stems are rich in xylem and phloem, this may result in the more abundant of puerarin in stems and roots relative to leaves.

The reason for distribution of puerarin in different tissues and varieties is attributed to the enzyme contents and activities involved in flavonoids metabolism. Therefore, a fuller understanding of the molecular mechanisms underlying the regulation of SG and TF genes will be of great importance to delineate the puerarin biosynthesis mediated by these enzymes. TFs participate in flavonoid biosynthesis processes by regulating the gene expression in plants [17]. It is well known that *R2R3-MYB* genes are extensively involved in a large of biological processes, especially involved in the regulation of secondary metabolism [28, 31, 45]. The phylogenetic tree including PtR2R3-MYB protein and AtR2R3-MYB protein clearly demonstrated that these genes could be divided into 34 subfamilies. This classification was further supported by the results of gene motifs and structure analyses. The gene expression patterns of S5, S6, and S7 subfamily members of *PtR2R3-tMYB* were significantly related to puerarin synthesis (Fig. 4), which was consistent with the function of S5, S6, and S7 subfamily members of AtR2R3-MYB in regulating the phenylpropanoid pathway [9]. *AtR2R3-MYB* subfamily S7 regulate flavonoid biosynthesis in all tissues and subfamily S6 form complexes with members of the WD40 and bHLH families to involved in the biosynthesis of proanthocyanidins and anthocyanins [9]. In addition, the *AtR2R3-MYB* subfamily S4 was transcriptional repressors of different branches of the phenylalanine metabolic pathway [18, 35]. In this study, *PtMYB139* belong to subfamily S4 and was highest expressed in leaves, which were contrary to those of puerarin accumulation level (Fig. 4). It is suggested *PtMYB139* is a transcriptional repressors that regulates puerarin biosynthesis. In the future, we can verify whether *PtMYB139* can regulate puerarin biosynthesis through more in-depth experiments. *PtMYB022* and *PtMYB141*, which belong to subfamily S13 that likely regulates the phenylpropanoid pathway, was negatively and positively regulates puerarin biosynthesis in ZG19 and ZG11, respectively.

Correlation analyses were conducted in order to investigate the association between puerarin content and *PtR2R3-MYBs*, SGs in this study. The different correlation coefficient among puerarin content and expression level of different genes resulted mainly from the different backgrounds of *P. thomsonii*. Among the 13 genes in the cluster G, 10 genes were significantly correlated with puerarin accumulation patterns in different varieties and tissues (Fig. 5b, *r* > 0.50), indicating that members of cluster G may be the essential genes regulating puerarin biosynthesis. These major SGs of cluster G were located downstream of flavonoids biosynthesis, this phenomenon does not mean that other SGs have no role in puerarin biosynthesis. It might be caused by the complexity of puerarin biosynthesis through multiple enzymatic reactions and the lag of puerarin accumulation in upstream enzymatic reactions. Previous studies identified PlUGT43 as an enzyme responsible for the C- glucosylation of daidzein to puerarin in *P. lobate* [43]. The amino acid sequence of *PtUGT8* was 96.52% identical to that of *PlUGT43*, but the encoded protein does not have the same function as PlUGT43 in *P. thomsonii* [8]. The amino acid sequence of *PtUGT010* (Table S8) was 94.7 and 96.5% identical to that of *PlUGT43* and *PtUGT8*, respectively. Non-synonymous mutations of *PlUGT43* and *PtUGT8* alter the functions of the coding genes. The gene expression pattern of *PtUGT010* was significantly associated with puerarin content in ZG19 (*r* = 0.94, Table S12), but not in ZG11 (*r* = − 0.29, Table S10) and ZG39 (*r* = 0.26, Table S11). Are there non-synonymous mutations in the *PtUGT010* genes of ZG11, ZG19, and ZG39? Whether the non-synonymous mutation of *PtUGT010* changes its regulation mode of puerarin accumulation in different varieties needs further studies and verification.

**Fig. 9:**
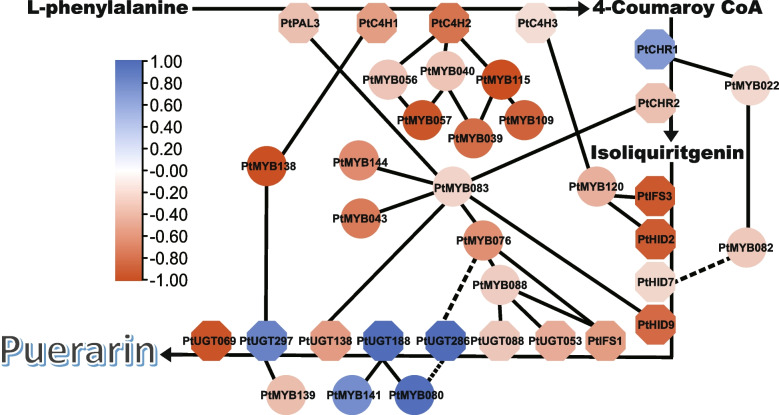
Illustration of the roles of *PtR2R3-MYBs* and SGs in regulation of puerarin biosynthesis in ZG11. The direct regulatory potential is connected with realization, and the indirect regulatory potential is connected with dotted lines. Circular, *PtR2R3-MYB* genes; octagon, SGs. The color scale is shown on the left, blue represents high correlation, red represents low correlation between puerarin content and gene expression level in ZG11

In addition, we determined the relationship between *PtR2R3-MYBs* and SGs by analyzing the correlation between the expression levels of their in different tissues and varieties, under MeJA treatment and GSH treatment (Fig. 8). And through the analysis of cis-acting elements to determine its direct or indirect regulatory relationship (Fig. 7). Therefore, we propose a functional model of *PtR2R3-MYBs* and SGs regulating puerarin biosynthesis in ZG11 (Fig. 9). *PtMYB039*, *PtMYB039*, *PtMYB109* indirect regulation upstream of puerarin biosynthesis by regulating MYB, while *PtMYB115* directly regulates the expression of *PtC4H2*. *PtMYB138* simultaneously regulates the expression of *PtC4H1* and *PtUGT069*. *PtUGT188* and *PtUGT286*, two key genes regulating puerarin biosynthesis, was directly regulated and indirectly regulated by *PtMYB141*, *PtMYB080* and *PtMYB076*, *PtMYB080*, respectively. Another key gene that regulates puerarin biosynthesis is directly negatively regulated by *PtMYB139*. The predictions of this model could be further tested through future experimental work.

### *PtR2R3-MYB* genes play crucial roles in hormone regulation of puerarin biosynthesis

Exogenous hormone promote the production of secondary metabolites in plants, such as ABA, JA, MeJA, MeJA, GSH [1, 25, 29, 42, 49]. Exogenous MeSA could increase flavonoid concentration in tea leaves [25]. ABA has been reported to promote the biosynthesis of flavonols, quercetin and kaempferol in grape [1]. It has also been reported that ABA and MeJA promote anthocyanin accumulation in Arabidopsis [29]. The identification of cis-elements showed that 44 candidate genes have at least one of the ABRE, ERE, JAE, CGTCA-motif, TGACG-motif, GARE-motif, P-box, TATC-box, TCA and TGA-elements, which are mainly involved in responses to hormone (Fig. 7 and Table S13). All 44 candidate genes were induced or repressed after MeJA and GSH treatment and showed different expression patterns, suggesting that they may play diverse roles in response to MeJA and GSH. A total of 27 candidate genes were found to have MeJA responsiveness elements, including CGTCA-motif and TGACG-motif. Recently, MeJA has been reported to promote the biosynthesis of puerarin [11, 26]. This indicates that 27 candidate genes maybe involved in regulation of puerarin biosynthesis by MeJA. It is worth noting that after MeJA and GSH treatment for 12–24 h, the expression changes of most candidate genes were consistent with the correlation of puerarin biosynthesis, which indicated that MeJA and GSH have the potential to mediate puerarin biosynthesis by regulating gene expression in ZG11.

## Conclusions

In present study, a total of 209 *PtR2R3-MYB* genes were identified. Furthermore, the physical and chemical characteristics, subcellular localization, gene structure, conserved motif, chromosomal location, phylogenetic relationships were performed. We determined the tissue expression patterns of *PtR2R3-MYBs* and SGs in different cultivars and expression changes under MEJA and GSH treatment by RNA-seq and qRT-PCR. In addition, we analyzed promoter cis-acting elements of candidate genes regulating puerarin biosynthesis. Overall, this study provides a comprehensive understanding of the *PtR2R3-MYB* and will paves the way to reveal the transcriptional regulation of puerarin biosynthesis and response to hormone of *PtR2R3-MYB* genes in *P. thomsonii*.

## Materials and methods

### Plant growth and stress treatments

Three *P. thomsonii* varieties with different puerarin properties (ZG11, ZG39, ZG19) were grown at scientific and technological demonstration park, GXAAS, Wuming Distract, Nanning, Guangxi, China. Fresh roots, stems, leaves were collected 10-month after planting, and were minced, mixed separately and divided in two samples; one sample was used to quantify puerarin content and the other one was immediately frozen in liquid nitrogen and used for RNA extraction.

Tissue cultured plantlets of ZG11 were transplanted into nutrition soil: coconut bran: perlite: sand (2:2:2:1:, v/v/v/v) mixture, then cultivate it at an artificial climate box (temperature, 26 °C; light intensity, 30,000 Lx; 14 h of light and 10 h of darkness) for 30 days. After washing away soil mixture, ZG11 were transferred to ddH_2_O containing 100 μmol/L MeJA or GSH. The treated ZG11 were incubated under continuous white light at 26 °C. Root samples were randomly collected from ten pots at 0, 6, 12, 24 h after treatment. At each time point, three biological replicates were taken. The collected roots samples were immediately frozen in liquid nitrogen and stored at − 80 °C until analyzed.

### Genime-wide identification of MYB genes in *P. thomsonii*

The whole-genome sequencing result data and annotation files of *P. thomsonii* ZG11 from NCBI (https://ftp.ncbi.nlm.nih.gov/genomes/all/GCA/019/096/045/GCA_019096045.1_GAAS_Ptho_1.0/) were downloaded. The Hidden Markov Model (HMM) profiles of MYB domain (PF00249) were downloaded from the Pfam database (http://pfam.xfam.org/). To find putative MYB family member of *P. thomsonii*, we first searched MYB domains from *P. thomsonii* protein database by using TBtools [2]. Then all putative *MYBs* of *P. thomsonii* genes were further verified from Pfam database with e-value cut-off at 1.0 by performing a Batch search and the incorrect and redundant predicted sequences were manually removed. The sequences containing two MYB-binding domain repeats were identified as *R2R3-MYB* of *P. thomsonii*. The copy number of *PtR2R3-MYB* genes were verified by multiple sequence alignment of full-length CDS sequences of *P. thomsonii* genome by TBtools [2]. Furthermore, BLAST comparison were performed by submitting the identified R2R3-MYB genes into the NCBI database (https://www.ncbi.nlm.nih.gov/).

The physical and chemical characteristics of PtR2R3-MYB proteins like amino acids length, molecular weight (MW) and isoelectric point (pI) were calculated by ProtParam tool in the EcPASy Server (https://web.expasy.org/protparam/). All the amino acid sequences of PtR2R3-MYB proteins were submitted to the online tool MEME (https://meme-suite.org/meme/tools/meme), where we performed motifs analysis with setting the maximum number of motifs to 10, other parameters were kept as default. The conserved motif and MYB-binding domains were visualized by TBtools [2].

### Chromosomal location, duplication modes and phylogenetic analysis of *PtR2R3-MYB* genes

The locations of *PtR2R3-MYB* loci on the chromosome was obtained from the genome annotation files [38]. The name of putative R2R3-MYB genes were encoded according to the physical location information of chromosomes. The distribution of *PtR2R3*-*MYBs* on the chromosome was mapped and the gene density was calculated and outputted by TBtools [2]. In addition, we identified different modes of gene duplication as WGD, TD, PD (less than 10 gene distance on the same chromosome), TRD, or DSD (other duplicates than WGD, TD, PD and TRD) using DupGen_finder with default parameters [36]. The Ka (non-synonymous rate), Ks (synonymous rate) and Ka/Ks ratios were calculated by using Simple Ka/Ks Calculator (NG) program of TBtools according to their CDS sequence [2]. The duplication time was calculated according to published method by using the following formula: Time = Ks/(2 × 5.17 × 10^− 3^) Million years [38].

The amino acid sequences of 126 R2R3-MYB genes of *A. thaliana*, as described before [9], were downloaded from the Plant Transcription Factor Database (http://planttfdb.gao-lab.org/). All the R2R3-MYB sequences were aligned using Clustal X 2.0 [41], and a Maximum Likelihood phylogenetic tree was constructed with 1000 bootstrap replicated utilizing MEGA X [21].

### Determination of puerarin content by HPLC method

Fresh roots, stems, leaves of ZG11, ZG19, ZG39 were weighed, oven-dried at 75 °C to a constant weight, and weighed again. Dried samples (0.1 g) was ground to a fine powder and extracted twice with 2.5 ml of pre-cooled 50% methanol by ultrasonic at 25 °C for 30 min. The solution was centrifuged at 12,000 × rpm for 10 min at room temperature, take the supernatant to volume to 5 mL with 50% methanol. The supernatant was filtered through a 0.22 μm filter and used for HPLC analysis.

The content of puerarin was measured using HPLC. HPLC analysis was performed using a LC-100 instrument (Wufeng, China) equipped with an C18 column (250 mm × 4.6 mm, 5 μm). The detection was monitored at 250 nm. The mobile phase composed of solvent A (100% methanol) and solvent B (water) at a volume ratio of 55:45. The HPLC injection volume was 10 μL, flow rate was 0.8 mL/min and total run time was 25 min. Column temperature was maintained at 30 °C. The linear range of puerarin was 0.5–10,000 μg/mL, Y = 48.677X-1.9149 (*r*^2^ = 0.9990). The limits of quantification (LOQ) was 0.5 μg/mL.

### RNA library construction and transcriptome sequencing

Total RNAs of roots, stems, leaves of ZG39, ZG19 were extracted using HiPure HP Plant RNA Mini Kit (Magen, China) according to the manufacturer’s instructions. For each sample, an equal amount (1 μg) of total RNA was used to generate transcriptome libraries by Illumina mRNA-seq Library Preparation kit for RNA sequencing. RNA sequencing was performed on the Illumina HiSeq 2000 platform with 150 bp paired-end reads sequencing.

Adaptor sequences and low quality reads (reads having more than 5% unknown nucleotides or with Q20 lower than 20%) were filtered. The filtered clean reads can be found in the NCBI SRA repository (https://www.ncbi.nlm.nih.gov/sra), with the accession No. PRJNA723378 [38]. The clean reads were mapped to *P. thomsonii* genome by HISAT2 [20]. Read mapping and transcript abundance quantification (FPKM-normalized expression value) were performed using Bowtie2 [22] and RSEM [24]. Differential expression analysis of unigenes was completed through DEseq2 (version 1.22.2) [30], with a threshold of |log2(fold change)| ≥1 and the false discovery rate (FDR) adjusted *p*-value lower than 0.05. The *PtR2R3-MYB* expression values were calculated by log2 (FPKM) and displayed as a heat map generated using heatmap program of TBtools [2].

### Gene expression analysis (RT-qPCR)

To validate the gene expression profiles identified by RNA-seq, total RNA of roots, stems, leaves of ZG11, ZG19, ZG39 and roots after MeJA or GSH treatment 0, 6, 12, 24 h of ZG11 were extracted as above. CDNA was synthesized using the HiScript® II Q RT SuperMix for qPCR (+gDNA wiper) Kit (Vazyme, China). Gene specific primers were designed using Primer Premier 5 (http://www.premierbiosoft.com/primerdesign/), Geneious Pro 4.8.5 (http://www.geneious.com/) and tested for specificity by using TBtools [2]. Primers were designed to have amplicon lengths of 80–250 bp, GC contents of 40–60% and Tm values of 55–65 °C (Table S16). *PtGAPDH* (Pmon010G03453) was used as the housekeeping gene. Quantitative RT-PCR was performed using TB Green® Premix Ex Taq™ II (Tli RNaseH Plus) (TAKARA, China) on the LightCycler® 480 instrument (Roche, Germany) with the following conditions: 95 °C for 30s and then 45 cycles of 95 °C for 5 s and 55–65 °C for 20s, followed by a melt cycle of 65 °C for 5 s and 95 °C for 15 s. All qRT-PCR experiments were taken in three biological replicated and each reactions were performed in triplicate. The relative expression of each gene was calculated with the 2^-△△Ct^ methods and the values were the means± SEMs.

### Correlation analysis

The correlation analysis of the puerarin contents and *PtR2R3-MYB* genes, SGs were identified in the “corrplot” package of R software (version 4.1.2, https://www.r-project.org/). The correlations heatmap of *PtR2R3-MYB* genes and SGs were made using the normalized method by heatmap program of TBtools [2].

### Analysis of cis-regulatory elements

A 2 kb region upstream of each gene start codon was extracted to predict putative cis-element of *PtR2R3-MYB* genes and SGs by PlantCARE database [23]. The elements involved in MYB DNA binding site and hormone response were summarized.

## Supplementary Information

**Additional file 1: Table S1.** MYB-binding domain of *PtMYBs*. **Table S2.** Details information of the identified *PtR2R3-MYB* genes. **Table S3.** The prediction of subcellular localization in PtR2R3-MYB proteins. **Table S4.** Duplication modes and Ka/Ks ratios of *R2R3-MYB* gene pairs in *P. thomsonii*. **Table S5.** Putative functions of the R2R3-MYB proteins in *P. thomsonii* and Arabidopsis. **Table S6.** Detailed information for the 10 motifs in the R2R3-MYB proteins of *P. thomsonii*. **Table S7a.** Correlation between puerarin contents, SGs, *PtR2R3-MYBs* by RNA-seq data in *P. thomsonii*. **Table S7b.** *P* values between puerarin contents, SGs, PtR2R3-MYBs by RNA-seq data in P. thomsonii. **Table S8.** Structural genes related to puerarin biosynthesis. **Table S9a.** Correlation between puerarin and 46 genes by qRT-PCR data in 9 samples. **Table S9b.** *P* values between puerarin and 46 genes by qRT-PCR data in 9 samples. **Table S10a.** Correlation between puerarin and 46 genes by qRT-PCR data in ZG11. **Table S10b.** *P* values between puerarin and 46 genes by qRT-PCR data in ZG11. **Table S11a.** Correlation between puerarin and 46 genes by qRT-PCR data in ZG39. **Table S11b.** *P* values between puerarin and 46 genes by qRT-PCR data in ZG39. **Table S12a.** Correlation between puerarin and 46 genes by qRT-PCR data in ZG19. **Table S12b.** *P* values between puerarin and 46 genes by qRT-PCR data in ZG19. **Table S13.** Prediction of cis-acting elements in the promoter regions of 44 genes. **Table S14a.** Correletion between 44 genes qRT-PCR data by GSH treatment in ZG11. **Table S14b.** *P* values between 44 genes qRT-PCR data by GSH treatment in ZG11. **Table S15a.** Correletion between 44 genes qRT-PCR data by MeJA treatment in ZG11. **Table S15b.** *P* values between 44 genes qRT-PCR data by MeJA treatment in ZG11. **Table S16.** Primer sequence for qRT-PCR. **Table S17.** Puerarin contents and gene expression level by qRT-PCR.

## Data Availability

The transcriptome sequencing data can be found in SRA database of NCBI repository under the umbrella of BioProject Accession PRJNA723378.

## Abbreviations

*P. thomsonii*: *Pueraria lobata var. thomsonii*
*P. lobate*: *Pueraria lobate*
*A. thaliana*: *Arabidopsis thaliana*
PAL: Phenylalanine ammonialyase;
C4H: Cinnamate 4-hydroxylase
4CL: 4-coumarate-CoA ligase
CHS: Chalcone synthase
CHI: Chalcone isomerase
CHR: Chalcone reductase
IFS: 2-hydroxyisoflavanone
HID: 2-hydroxyisoflavanone dehydratase
UGT: UDP-glucosyltransferases
SG: Structural gene
TF: Transcription factors
WGD: Whole-genome duplication
TD: Tandem duplication
PD: Proximal duplication
DSD: Dispersed duplication
TRD: Transposed duplication
